# Leaf wax *n‐*alkane patterns of six tropical montane tree species show species‐specific environmental response

**DOI:** 10.1002/ece3.5458

**Published:** 2019-07-21

**Authors:** Milan Lana Teunissen van Manen, Boris Jansen, Francisco Cuesta, Susana León‐Yánez, William Daniel Gosling

**Affiliations:** ^1^ Department of Ecosystem and Landscape Dynamics, Institute for Biodiversity and Ecosystem Dynamics (IBED) University of Amsterdam (UvA) Amsterdam The Netherlands; ^2^ Grupo de Investigación en Biodiversidad, Medio Ambiente y Salud (BIOMAS) Universidad de Las Américas (UDLA) Quito Ecuador; ^3^ Escuela de Ciencias Biológicas Pontificia Universidad Católica de Ecuador (PUCE) Quito Ecuador

**Keywords:** Ecuador, leaf wax, lipid biomarkers, *n*‐alkanes, species‐specific response, Western Andes

## Abstract

It remains poorly understood how the composition of leaf wax *n*‐alkanes reflects the local environment. This knowledge gap inhibits the interpretation of plant responses to the environment at the community level and, by extension, inhibits the applicability of *n‐*alkane patterns as a proxy for past environments. Here, we studied the *n‐*alkane patterns of five *Miconia* species and one *Guarea* species, in the Ecuadorian Andes (653–3,507 m a.s.l.). We tested for species‐specific responses in the average chain length (ACL), the C_31_/(C_31_ + C_29_) ratio (ratio), and individual odd *n‐*alkane chain lengths across an altitudinally driven environmental gradient (mean annual temperature, mean annual relative air humidity, and mean annual precipitation). We found significant correlations between the environmental gradients and species‐specific ACL and ratio, but with varying magnitude and direction. We found that the *n*‐alkane patterns are species‐specific at the individual chain length level, which could explain the high variance in metrics like ACL and ratio. Although we find species‐specific sensitivity and responses in leaf *n*‐alkanes, we also find a general decrease in “shorter” (<C_29_) and an increase in “longer” (>C_31_) chain lengths with the environmental gradients, most strongly with temperature, suggesting *n*‐alkanes are useful for reconstructing past environments.

## 1 INTRODUCTION

Fully understanding the way in which plant leaf waxes reflect their environment could give an insight into plant resilience to future climate change (Guo, Guo, He, & Gao, 2015) and is instrumental for reconstructing past environmental conditions (Jansen & Wiesenberg, 2017). The *n‐*alkane fraction of the leaf wax, typically in the range of C_20_ and C_37_ (Eglinton & Hamilton, 1967), in particular has been suggested as a proxy for past environments due to their resistance to degradation (Jansen & Wiesenberg, 2017). Studies measuring *n‐*alkane patterns across plant communities, functional groups, genera, and species have shown that the *n‐*alkane pattern seems to be influenced by several environmental factors, notably temperature and precipitation (Bush & McInerney, 2013, 2015; Feakins et al., 2016; Hoffmann, Kahmen, Cernusak, Arndt, & Sachse, 2013; Sachse, Radke, & Gleixner, 2006; Tipple & Pagani, 2013). However, it has been hard to identify a general trend in the *n‐*alkane signal in response to the environmental factors.

There have been numerous works exploring *n‐*alkane patterns at the plant community or functional group level (Bush & McInerney, 2015; Feakins et al., 2016), but there are fewer studies at lower taxonomic levels (Hoffmann et al., 2013; Maffei, Mucciarelli, & Scannerini, 1993; Sachse et al., 2006; Tipple & Pagani, 2013). Within the studies done at lower taxonomic levels, the results are mixed; one study finds a genus specific response to hydrological factors but not to temperature (Hoffmann et al., 2013), while two other studies find species‐specific response to increasing temperature (Sachse et al., 2006; Tipple & Pagani, 2013). With limited knowledge on leaf wax *n‐*alkanes at lower taxonomic levels, it remains unclear how prevalent these species‐specific responses are and what characterizes them.

In this study, we aim to further the knowledge of species‐specific responses in leaf wax *n‐*alkane patterns. Specifically, we ask whether we observe species‐specific responses in the *n‐*alkane patterns from six tropical tree species, sampled along an altitudinally driven, environmental gradient in Ecuador.

### 1.1 Study region and species selection

**Figure 1:**
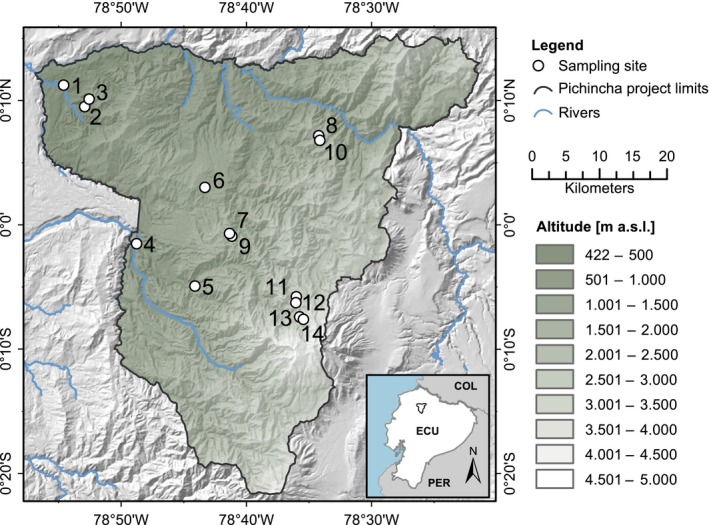
Map of the Pichincha transect. Dots and numbers refer to sites in Appendix S1, ordered by altitude. The green shading indicates altitude (m above sea level). The black delineates the Pichincha project study area. Blue lines represent rivers, land codes as follows: COL = Colombia, ECU = Ecuador, PER = Perú

The area of study is the north western flank of the Andes (Pichincha province, Ecuador) (Figure 1). This study took advantage of the Pichincha long‐term forest development and carbon monitoring transect that was established in 2015 by the research nonprofit organization Consorcio para el Desarrollo Sostenible de la Ecorregión Andina (CONDESAN) (http://condesan-ecoandes.org/). CONDESAN catalogued the tree community composition and recorded environmental data at each site along the transect (Pinto, Pérez, Ulloa Ulloa, & Cuesta, 2018). The transect captures environmental gradients in mean annual temperature (7.2–21.6°C), relative air humidity (96.1%–99.8%), and mean annual precipitation (1,580–2,448 mm; Karger et al., 2017).

To investigate species‐specific responses in leaf wax *n‐*alkanes, we targeted genera and species that were abundant and had a wide distribution along the Pichincha transect. We selected the most abundant and widely distributed species in the area: five species of *Miconia* and one species of *Guarea* (Appendix S1). The genus *Miconia* is one of the most diverse, widely distributed, genera found in the Ecuadorian Andes (Jørgensen & León‐Yánez, 1999) and spans the entire transect (2,854 m, 100% of transect length). *Guarea kunthiana* is an especially ubiquitous species in northern Ecuador (Jørgensen & León‐Yánez, 1999) and is the most widely distributed single species on the altitudinal transect (1559 m, 55% of transect length).

## 2 MATERIALS AND METHODS

### 2.1 Sampling and environmental data collection

A total of 87 samples (one sample per individual) were collected along the Pichincha transect between 500 and 2,500 m a.s.l, from 14 permanent vegetation study sites. We aimed to sample at least three individuals per species per site, but this was not always possible as species were not always present, or abundant, at every site (Appendix S1).

Each sample consists of 20–25 leaf(lets) collected from the canopy. We selected undamaged leaf(lets) and a wide range of leaf sizes and ages (Koch & Ensikat, 2008). Samples were collected using gloves and were wrapped in aluminum foil in the field, making sure no contact was made with the skin to avoid lipid contamination. Samples were bagged and placed in a cold storage (5°C) until further analysis at the University of Amsterdam.

Temperature and relative air humidity were measured at each site using Onsetcomp HOBO U23 Pro v2 Temperature/Relative Humidity data loggers at an hourly basis from 2016 to 2018. We calculated mean annual temperature (hereafter “temperature”) and mean relative air humidity (hereafter “humidity”) per site based on the two‐year dataset. Mean annual precipitation (Bioclim 12, hereafter “precipitation”) per site was obtained from the CHELSA dataset at 30‐S resolution (1 km) (Karger et al., 2017).

### 2.2 Leaf wax alkane extraction and analysis

Lipid extraction was done following the protocol described in Jansen et al. (2013) with minor modifications. Leaf samples were freeze‐dried and milled to powder prior to analysis, and 0.1 g of the milled sample was extracted by accelerated solvent extraction (ASE) using a Dionex 200 ASE extractor. ASE was done at 75°C and 1,500 pKa employing a heating phase of 5 min and a static extraction time of 20 min. Dichloromethane/methanol (DCM/MeOH) (93:7 v/v) was used as the extractant. We added 60 µg of a mixture of androstane, androstan, and euric acid (0.3 µg/µl per compound) to each extraction to provide an internal standard.

Fractionation was done using a 10 ml solid phase column with approximately 1.5 g of silica gel (5% deactivated H_2_O) previously conditioned with acetone, DCM, and hexane. The *n‐*alkane fraction was obtained by eluting the column with hexane. The *n‐*alkane fraction was analyzed by injecting 1 µl of the sample into a GC‐MS with quadruplet MS detection in full scan mode (50–650 amu), conform the following protocol: The sample was injected in a DB5 column (30 m) under constant flow of gas helium 0.8 ml/min. Temperature programming was: first ramp 60°C/min to 80°C (hold 2 min), second ramp 20°C/min to 130°C, 4°C/min to 350°C (hold 10 min). *n‐*Alkanes were identified and quantified by comparing with a known mixture of *n‐*alkanes in the range C_25_–C_33_ and the internal standard employing the Thermo Xcalibur® software. We identified *n‐*alkanes between C_23_ and C_33_, all of which are known to contribute to the higher plant leaf wax *n‐*alkane fraction (Eglinton & Hamilton, 1967). The resulting *n‐*alkane dataset was standardized by sample weight (grams of dry leaf sample used for extraction) (hereafter, *n‐*alkane data).

Three samples from different species and altitudes were selected for replicate analysis (two samples run in triplicate and one sample in duplicate), and three other samples were already split in the field. In total, 14 replicate measurements were done simultaneous with the rest of the analysis in order to check measurement variability.

### 2.3 Overall *n*‐alkane patterns, metrics, and environmental correlations

To obtain species overall *n‐*alkane patterns along the transect, we standardized *n‐*alkane data to total *n‐*alkane concentration (hereafter, relative abundances) and then calculated species means and standard deviation from the mean.

In order to study shifts in the *n‐*alkane pattern along the environmental gradient, we correlated two commonly used metrics (weighted average chain length (ACL) and the C_31_/C_29_ ratio) and the relative abundances to temperature, humidity, and precipitation.

The ACL was calculated following Bush and McInerney (2013):

(1) $${\text{ACL}}_{21-33}=\Sigma \left(C_{n}\times n\right)/\left(\Sigma C_{n}\right)$$

where C_n_ is the concentration of *n‐*alkane per gram of dried sample, and *n* is the number of carbon atoms of the measured *n‐*alkane (C_21_–C_33_).

The ratio between C_31_ and C_29_ *n‐*alkanes was calculated following Bush and McInerney (2013):

(2) $$\text{ratio}=C_{31}/\left(C_{31}+C_{29}\right)$$

where C_31_ and C_29_ stand for the concentrations of the C_31_ and C_29_ *n‐*alkanes, respectively. We chose the ratio as a fraction of one because it is easy to interpret and it standardizes variances, as opposed to the simple fraction that is also reported in literature (Jansen & Nierop, 2009). Other ratios found in literature were also calculated, but those ratios behaved similar to the ratio presented here (Equation (2)) (Appendix S2) and thus are not included in further analysis. Two ratio values of *Miconia theaezans* and *Miconia clathrantha* were abnormally small compared to similar samples at the same site. We therefore removed these from all analysis (see open symbols in Figure 3).

We correlated the ACL, the ratio, and the relative abundances of the species sampled at three or more sites to temperature, humidity, and precipitation using Spearman's rank order correlation (i.e., “species response”). We also correlated the ACL, the ratio, and relative abundances of the entire dataset (of all six species) to temperature, humidity, and precipitation using Spearman's rank order correlation (i.e., “site total response”).

We chose to only show the correlations with the relative abundances of the odd chain lengths because these dominate the higher plant *n‐*alkane signal (Eglinton & Hamilton, 1967). We adopted a significance level of *p*‐value <.01 because the bulk of our analysis is correlations (following convention, e.g., Tipple & Pagani, 2013). Additionally, it should be noted that the environmental gradients are sampled across an elevational gradient, and therefore, the environmental variables are not independent (Appendix S3). All statistical analyses were performed in R studio using the base statistics functions (R Core Team, 2017) and the “tidyverse” package functions (Wickham, 2017).

## 3 RESULTS

### 3.1 Overall *n*‐alkane patterns

Replicate measurements showed high coefficients of variance for absolute *n‐*alkane concentration (most samples >10%, Appendix S4). However, the metrics had very low coefficients of variance (ACL below 0.5%, ratio below 9%, Appendix S4), giving us confidence that the relative measurements and metrics are accurate.

**Figure 2:**
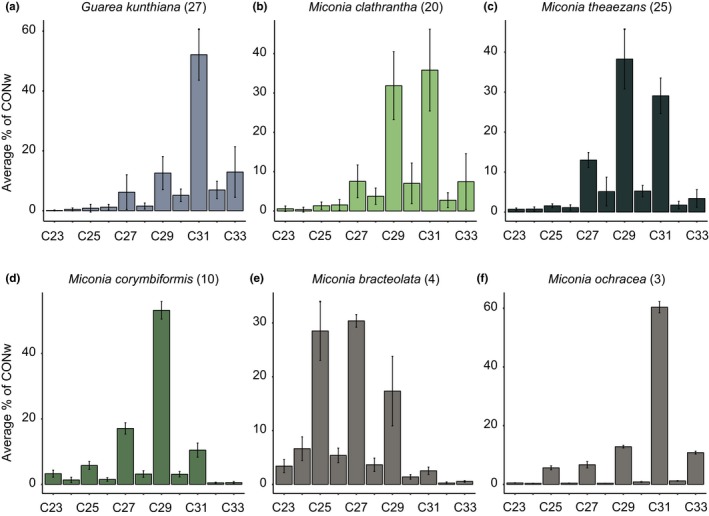
Species average *n‐*alkane distributions (a–f), showing average relative abundances (average % of total concentration (CONw)) across entire transect. Lines represent standard deviation from average; numbers in parentheses represent number of measurements (excluding outliers)

The average species *n‐*alkane pattern is typical of the leaf wax fraction of higher plants (Figure 2). All species have an odd‐over‐even preference (OEP), typical of plants (Eglinton & Hamilton, 1967). Only the C_23_ chain length deviated from the OEP in three of the six species, namely *G. kunthiana*, *M. theaezans,* and *Miconia bracteolata* (Figure 2).

### 3.2 Average chain length (ACL)

**Figure 3:**
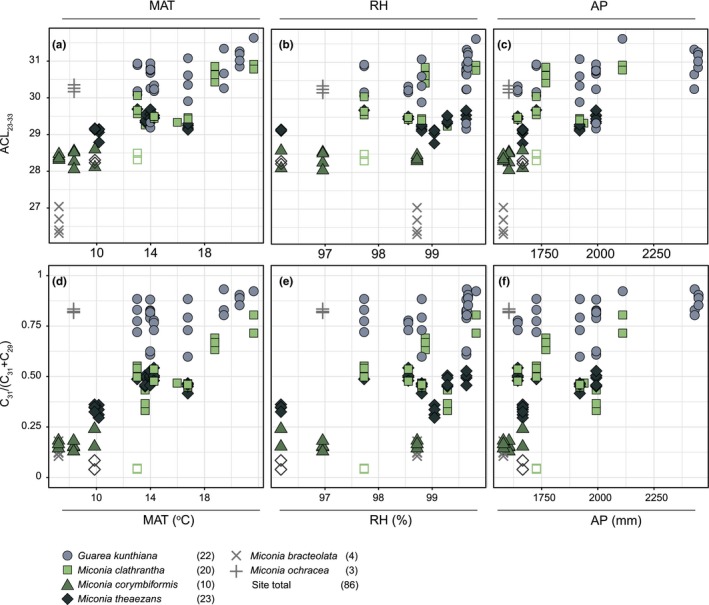
(a–c) Average chain length (ACL_23–33_) and (d–f) the ratio (C_31_/(C_31_ + C_29_)) distribution along three environmental gradients: (a,d) mean annual temperature (MAT), (b,e) mean relative air humidity (RH), and (c,f) mean annual precipitation (AP). Spearman's rank correlation coefficients and significance are in Figure 4 and Appendix S5. Colors and symbols correspond to species. Open symbols indicate outliers that were excluded from all analysis. Gray symbols (×, +) indicate species sampled at less than three sites. Site total includes data points of all species, excluding outliers. Numbers in parentheses indicate number of data points per correlation

**Figure 4:**
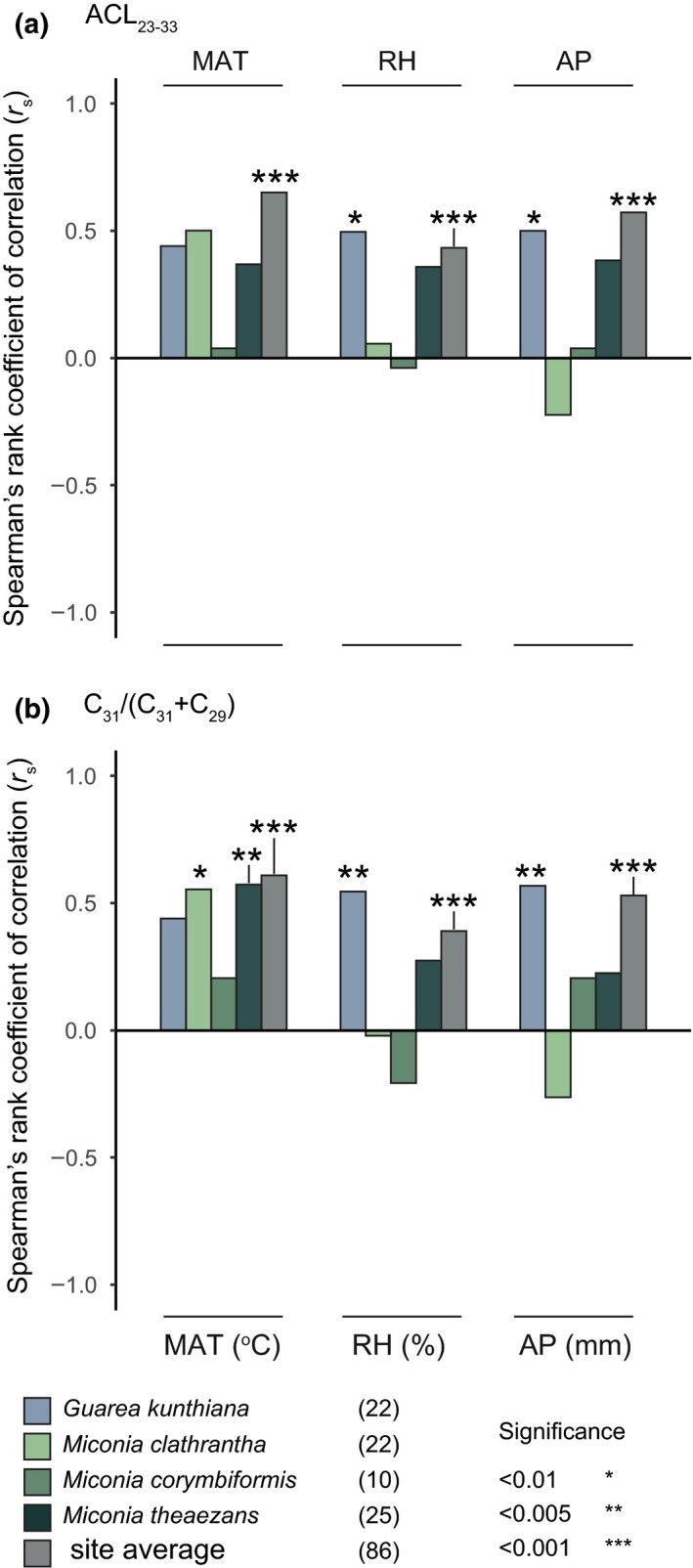
Spearman's rank correlation coefficients of (a) average chain length (ACL_23–33_) and (b) ratio (C_31_/(C_31_ + C_29_)) from individual species and site total against three environmental gradients: mean annual temperature (MAT), mean relative air humidity (RH), and mean annual precipitation (AP). Colors correspond to species (blue and greens) and site total (gray). Numbers in parentheses indicate number of data points per correlation

The ACL of one species and the site total significantly correlated with the environmental variables (Figures 3a–c and 4a, Appendix S5). Specifically, humidity and precipitation correlated positively with the ACL of *G. kunthiana* (*r*_s_ = .5, *p* = .008 and *r*_s_ = .5, *p* = .007, respectively). *Miconia corymbiformis* ACL was not significant for any of the environmental variables, but this is most likely due to the small sample size and short gradient over which it was sampled (Appendix S1). Site total ACL correlated positively with all environmental variables (Figures 3a–c and 4a, Appendix S5; temperature: *r*_s_ = .66, *p* < .001, humidity: *r*_s_ = .44, *p* < .001, and precipitation: *r*_s_ = .57, *p* < .001).

### 3.3 Ratio

We observed four significant species‐specific correlations with the environmental gradient (Figures 3d‐f and 4b, Appendix S5). Temperature positively correlated with the ratio of *M. clathrantha* and *M. theaezans* (*r*_s_ = .56, *p* = .009, *r*_s_ = .58, *p* = .003, respectively. Figures 3d and 4b, Appendix S5). *G. kunthiana* correlated significantly with humidity and precipitation (Figure 4e,f, Appendix S5; humidity: *r*_s_ = .55, *p* = .003, precipitation: *r*_s_ = .57, *p* = .002). The ratio of *M. corymbiformis* was not significant for any of the environmental variables, likely due to the limited sampling (Appendix S1). The site total ratio correlated positively with all environmental variables (Figures 3d–f and 4b, Appendix S5; temperature: *r*_s_ = .61, *p* < .001, humidity: *r*_s_ = .39, *p* < .001, and precipitation: *r*_s_ = .53, *p* < .001).

#### 3.3.1 Odd chain species‐specific *n*‐alkane percentages

**Figure 5:**
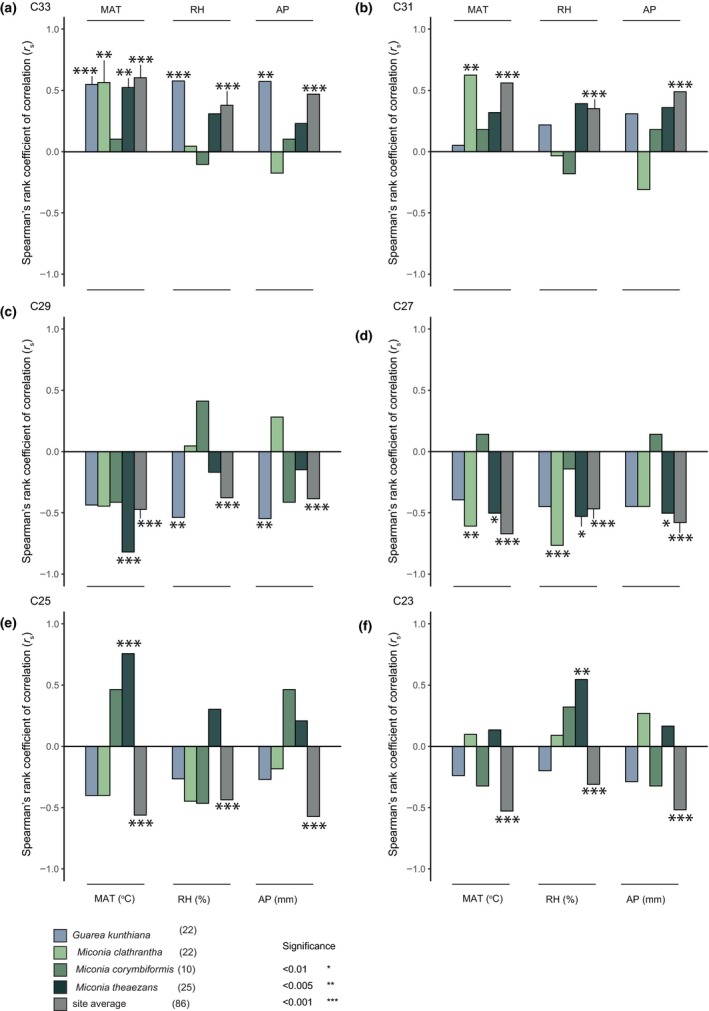
Spearman's rank correlation coefficients of odd chain length *n‐*alkanes (a–f) from individual species and site total against three environmental gradients: mean annual temperature (MAT), mean relative air humidity (RH), and mean annual precipitation (AP). Colors correspond to species (blue and greens) and site total (gray). Numbers in parentheses indicate number of data points per correlation

Correlations between the environmental gradients and the percentages of odd chain *n‐*alkanes varied between chain lengths and species (Figure 5, Appendix S6).

Temperature and relative abundances of C_33_ significantly correlated for all species, except *Miconia corymbiformis* (due to limited sampling) (Figure 5a, Appendix S6). Furthermore, the C_31_ fraction of *M. clathrantha* correlated with temperature (*r*_s_ = .63, *p* = .002 Figure 5b). The C_29_ fraction of *M. theaezans* correlated negatively with temperature (*r*_s_ = −.82, *p* < .001, Figure 5c, Appendix S6). The C_27_ fraction of *M. clathrantha* and *M. theaezans* correlated negatively with temperature (*r*_s_ = −.61, *p* = .003 and *r*_s_ = .51, *p* = .010, respectively; Figure 5d, Appendix S6). Additionally, the C_25_ fraction of *M. theaezans* significantly correlated with temperature (*r*_s_ = .76, *p* < .001; Figure 5e, Appendix S6).

Humidity correlated significantly with the relative abundances of *G. kunthiana*. Specifically, with increasing humidity the fraction of C_33_ increased, the fraction of C_29_ decreased (*r*_s_ = .58, *p* = .002 and *r*_s_ = −.54, *p* = .004, respectively; Figure 5a,c, Appendix S6). Additionally, humidity and the C_27_ fraction of *M. clathrantha* also correlated negatively (*r*_s_ = −.77, *p* < .001). Contrastingly, the C_23_ fraction of *M. theaezans* correlated positively with humidity (*r*_s_ = .55, *p* = .004; Figure 5f, Appendix S6). Precipitation significantly correlated with the fraction of C_33_ and C_29_ in *G. kunthiana* (*r*_s_ = .58, *p* = .002 and *r*_s_ = −.52, *p* = .001, respectively; Figure 5a,c, Appendix S6).

At the site level, we observed that all environmental variables correlated positively with “longer” chain lengths (C_33_ and C_31_) and negatively with “shorter” chain lengths (C_29_, C_27_, C_25_, C_23_) (Figure 5a–d, Appendix S6).

## 4 DISCUSSION

### 4.1 Species‐specific responses to environmental gradients

We find that leaf wax *n‐*alkane distributions from the taxa studied here vary with the environmental gradient (Figure 2) and do so in species‐specific ways (Figure 3, Figure 4, and Figure 5).

#### 4.1.1 Temperature

Our results suggest that the *n‐*alkane fraction of *M. clathrantha* and *M. theaezans* are sensitive to temperature. However, the metrics differ in the way they reflect the shift in *n‐*alkane distributions (Figure 3, Figure 4). The underlying shifts in individual odd chain lengths indicate that the two *Miconia* species respond in distinct ways to the temperature gradient. In fact, the two species only overlap in the C_33_ and C_27_ chain lengths shifts with temperature (Figure 5). Shifts in the relative abundance of individual chain lengths have been observed before (Sachse et al., 2006); however, it is not known why species‐specific *n‐*alkane shifts occur at the individual chain length level. One reason for the contrasting distribution patterns and trends could be the large genetic diversity of the *Miconia* genus (Goldenberg et al., 2013; Goldenberg, Penneys, Almeda, Judd, & Michelangeli, 2008). Despite these species‐specific differences, the results also suggest that both *Miconia* species increase the relative abundance of “longer” chain lengths with increasing temperature, as highlighted by the ratio (Figure 3, Figure 5b,c). This indicates that the relationship between increased biosynthesis of “longer” chain *n‐*alkanes and temperature (Koch & Ensikat, 2008) is also at play here.

#### 4.1.2 Humidity and precipitation

Our results of both the ACL and ratio metrics suggest that the leaf wax *n‐*alkane fraction of *G. kunthiana* is sensitive to the hydrological factors (humidity and precipitation) (Figures 3 and 4, Appendix S5). Closer inspection of the *n*‐alkane composition shows that this is due to a shift in relative abundance toward “longer” chain lengths; both “shorter” *n*‐alkanes decrease and “longer” *n*‐alkanes increase (Figure 5a,c). The shifts in individual chain lengths with hydrological factors are pronounced in *Guarea,* but a less pronounced result is also seen in the *Miconia* species (Figure 5d,f). This suggests that not all species are equally sensitive or respond similarly. This falls in line with work done by Hoffmann et al., 2013, who found opposite correlations between hydrological factors (including humidity and precipitation) and the ACL of two genera in Australia. The reason for the difference in sensitivity is unknown and understudied to this point. However, Hoffmann et al., 2013 suggest leaf morphological and evolutionary differences could be important. Leaf morphology could also be playing a role in the differences observed between the two genera studied here—*Guarea* has large compound leaves, composed of paripinnate leaflets, whereas *Miconia* has simple leaves (Pinto, 2018).

### 4.2 Site total responses

Unlike the species metrics, all site total species‐specific *n‐*alkane metrics correlated positively with the environmental variables (Figures 3 and 4, Appendix S5).

The results in our study, the uniformity in the site total species‐specific *n‐*alkane shifts, were due to the positive correlation of ≥C_31_ (“longer” chain lengths, Figure 5a,b) and negative correlation of ≤C_29_ (“shorter” chain lengths, Figure 5c,d) with the environmental (temperature in particular). This suggests that, generally, the relative contribution of “longer” *n‐*alkanes increased with temperature, which falls in line with findings on plant communities and soils (Bush & McInerney, 2015; Feakins et al., 2016; Tipple & Pagani, 2013). It is, however, important to note that our data should not be viewed as a “community average” because it is based on a limited taxonomic sample and, as such, does not reflect the overall composition of the vegetation in our study plots. Further research is required to scale up our findings to the community level and establish whether the pattern is robust, specifically, by exploring the species‐specific *n‐*alkane pattern at the individual chain length level of plant communities and in soils.

## 5 CONCLUSION

Our results indicate that the leaf wax *n‐*alkane patterns in our study are determined by (a) species‐specific sensitivity to particular environmental gradients, and (b) species‐specific responses at the individual chain length level. We also note these species‐specific *n‐*alkane shifts are part of complex multivariate patterns that likely introduce variance into summarizing metrics, such as ACL or a ratio of individual chain lengths. Our results suggest that there is no “one size fits all” when it comes to characterizing leaf wax species‐specific *n‐*alkane responses. Alternative approaches, such as, looking at the relative abundances of individual chain lengths (e.g., Figure 5) or multivariate analysis (e.g., Maffei et al., 1993) might be more informative to characterize the *n*‐alkane fraction.

Despite the evidence for species‐specific responses at the chain length level, and the variance this likely introduces in summarizing metrics, we observe a general tendency of increasing relative abundances of “longer” *n‐*alkanes with the environmental gradients. This aligns with what has been found in plant communities and soils (Bush & McInerney, 2015; Feakins et al., 2016; Tipple & Pagani, 2013), suggesting the metrics and *n‐*alkane can be useful as a proxy for past environments.

Further research should focus on whether the results found here are applicable elsewhere, and on deconstructing typical species‐specific *n‐*alkane metrics (such as ACL) in order to uncover the underlying patterns and understanding variance. Additionally, it should be further investigated if species‐specific *n‐*alkane patterns from leaves reflect those extracted from soils. The degradation processes from leaf to sedimentary record remain understudied, having full understanding of this process is key to the application of *n‐*alkanes as a proxy for past environments.

## CONFLICTS OF INTEREST

None declared.

## AUTHOR CONTRIBUTIONS

MTVM, WDG, and BJ conceived and designed the study. MTVM, FCC, and SLY facilitated and conducted fieldwork. MTVM did the laboratory analysis and analyzed the data. MTVM, WDG, and BJ wrote the manuscript. All authors contributed to the drafts of the manuscript and its final approval.

## Data Availability

Environmental and *n‐*alkane data presented in this paper are accessible at Figshare https://doi.org/10.21942/uva.7610894
