# Supplementary material for: Leaf wax n‐alkane patterns of six tropical montane tree species show species‐specific environmental response
Source: Ecol Evol. 2019 Jul 21;9(16):9120–8. doi: 10.1002/ece3.5458 (PMC6706217; doi:10.1002/ece3.5458)
Supplement: Supplementary file 1 [file ECE3-9-9120-s001.docx]

| **Appendix 1** – Site metadata and sampling intensity. Site number corresponds with Figure 1. Three environmental gradients were recorded at each site: Mean annual temperature (MAT), mean relative air humidity (RH) and mean annual precipitation (AP). Total samples = Total number of samples taken at each site. Species sampled = names of the species sampled at each site, including its taxonomic reference and individuals = number of individuals per species sampled at each site. | | | | | | | | | | | | |  |
| --- | --- | --- | --- | --- | --- | --- | --- | --- | --- | --- | --- | --- | --- |
| **site nr** | **site** | **reserve** | **latitude** | **longitude** | **altitude** | **MAT** | **HR** | **AP** | **total samples** | **species sampled** | **species author and reference** | **individuals** | |
| 1 | MAPI 01 | Mashpishungo/Pambilina | 0.1873 | -78.9100 | 653 | 21.6 | 99.8 | 2075 | 2 | *Miconia clathrantha* | Triana ex Cogn. Monogr. Phan. 1891. | 1 | |
|  |  |  |  |  |  |  |  |  |  | *Guarea kunthiana* | A. Juss. Bull. Sci. Nat. Geol. 1830. | 1 | |
| 2 | MALO 01 | Mashpi Lodge | 0.1583 | -78.8819 | 827 | 19.4 | 99.6 | 2255 | 3 | *Guarea kunthiana* | A. Juss. Bull. Sci. Nat. Geol. 1830. | 3 | |
| 3 | MALO 02 | Mashpi Lodge | 0.1685 | -78.8761 | 1018 | 20.6 | 99.7 | 2253 | 3 | *Guarea kunthiana* | A. Juss. Bull. Sci. Nat. Geol. 1830. | 3 | |
| 4 | MIND 01 | Mindo Lindo | -0.0253 | -78.8129 | 1277 | 18.8 | 98.9 | 2704 | 4 | *Miconia clathrantha* | Triana ex Cogn. Monogr. Phan. 1891. | 4 | |
|  |  |  |  |  |  |  |  |  |  | *Miconia clathrantha* | Triana ex Cogn. Monogr. Phan. 1891. | 4 | |
| 5 | RIBR 01 | Reserva Rio Bravo | -0.0820 | -78.7353 | 1640 | 16.8 | 98.8 | 2347 | 12 | *Miconia theaezans* | (Bonpl.) Cogn. Fl. Bras. 1888. | 4 | |
|  |  |  |  |  |  |  |  |  |  | *Guarea kunthiana* | A. Juss. Bull. Sci. Nat. Geol. 1830. | 4 | |
| 6 | INTI 01 | Reserva Intillacta | 0.0505 | -78.7232 | 1879 | 16.0 | 98.8 | 2076 | 1 | *Miconia clathrantha* | Triana ex Cogn. Monogr. Phan. 1891. | 1 | |
| 7 | BECL 03 | Bellavista Cloud Forest | -0.0116 | -78.6893 | 2203 | 14.0 | 99.6 | 1595 | 8 | *Miconia theaezans* | (Bonpl.) Cogn. Fl. Bras. 1888. | 4 | |
|  |  |  |  |  |  |  |  |  |  | *Guarea kunthiana* | A. Juss. Bull. Sci. Nat. Geol. 1830. | 4 | |
|  |  |  |  |  |  |  |  |  |  | *Miconia clathrantha* | Triana ex Cogn. Monogr. Phan. 1891. | 4 | |
| 8 | CEDR 03 | El Cedral Ecolodge | 0.1132 | -78.5691 | 2212 | 14.3 | 98.6 | 1351 | 11 | *Miconia theaezans* | (Bonpl.) Cogn. Fl. Bras. 1888. | 4 | |
|  |  |  |  |  |  |  |  |  |  | *Guarea kunthiana* | A. Juss. Bull. Sci. Nat. Geol. 1830. | 3 | |
| 9 | BECL 01 | Bellavista Cloud Forest | -0.0153 | -78.6863 | 2313 | 13.6 | 99.3 | 1595 | 8 | *Miconia clathrantha* | Triana ex Cogn. Monogr. Phan. 1891. | 4 | |
|  |  |  |  |  |  |  |  |  |  | *Miconia theaezans* | (Bonpl.) Cogn. Fl. Bras. 1888. | 4 | |
|  |  |  |  |  |  |  |  |  |  | *Miconia clathrantha* | Triana ex Cogn. Monogr. Phan. 1891. | 4 | |
| 10 | CEDR 01 | El Cedral Ecolodge | 0.1195 | -78.5705 | 2492 | 13.0 | 97.7 | 1471 | 9 | *Miconia ochracea* | Triana.Trans. Linn. Soc. London. 1871[1872]. | 1 | |
|  |  |  |  |  |  |  |  |  |  | *Miconia theaezans* | (Bonpl.) Cogn. Fl. Bras. 1888. | 4 | |
| 11 | VERD 02 | Reserva Verdecocha | -0.1015 | -78.6004 | 2932 | 10.2 | 99.0 | 1251 | 4 | *Miconia theaezans* | (Bonpl.) Cogn. Fl. Bras. 1888. | 4 | |
| 12 | VERD 03 | Reserva Verdecocha | -0.1044 | -78.6008 | 3109 | 9.9 | 96.2 | 1251 | 6 | *Miconia corymbiformis* | Cogn. Bull. Acad. Roy. Sci. Belgique, sér. 3. 1887. | 2 | |
|  |  |  |  |  |  |  |  |  |  | *Miconia theaezans* | (Bonpl.) Cogn. Fl. Bras. 1888. | 4 | |
| 13 | VERD 01 | Reserva Verdecocha | -0.1233 | -78.5958 | 3421 | 8.3 | 97.0 | 1271 | 7 | *Miconia corymbiformis* | Cogn. Bull. Acad. Roy. Sci. Belgique, sér. 3. 1887. | 4 | |
|  |  |  |  |  |  |  |  |  |  | *Miconia ochracea* | Triana.Trans. Linn. Soc. London. 1871[1872]. | 3 | |
| 14 | YANA 01 | Reserva Yanacocha | -0.1267 | -78.5907 | 3507 | 7.2 | 98.7 | 1337 | 8 | *Miconia bracteolata* | (Bonpl.) DC. Prodr. 1828. | 4 | |
|  |  |  |  |  |  |  |  |  |  | *Miconia corymbiformis* | Cogn. Bull. Acad. Roy. Sci. Belgique, sér. 3. 1887. | 4 | |
