# Supplementary material for: Leaf wax n‐alkane patterns of six tropical montane tree species show species‐specific environmental response
Source: Ecol Evol. 2019 Jul 21;9(16):9120–8. doi: 10.1002/ece3.5458 (PMC6706217; doi:10.1002/ece3.5458)
Supplement: Supplementary file 2 [file ECE3-9-9120-s002.pdf]

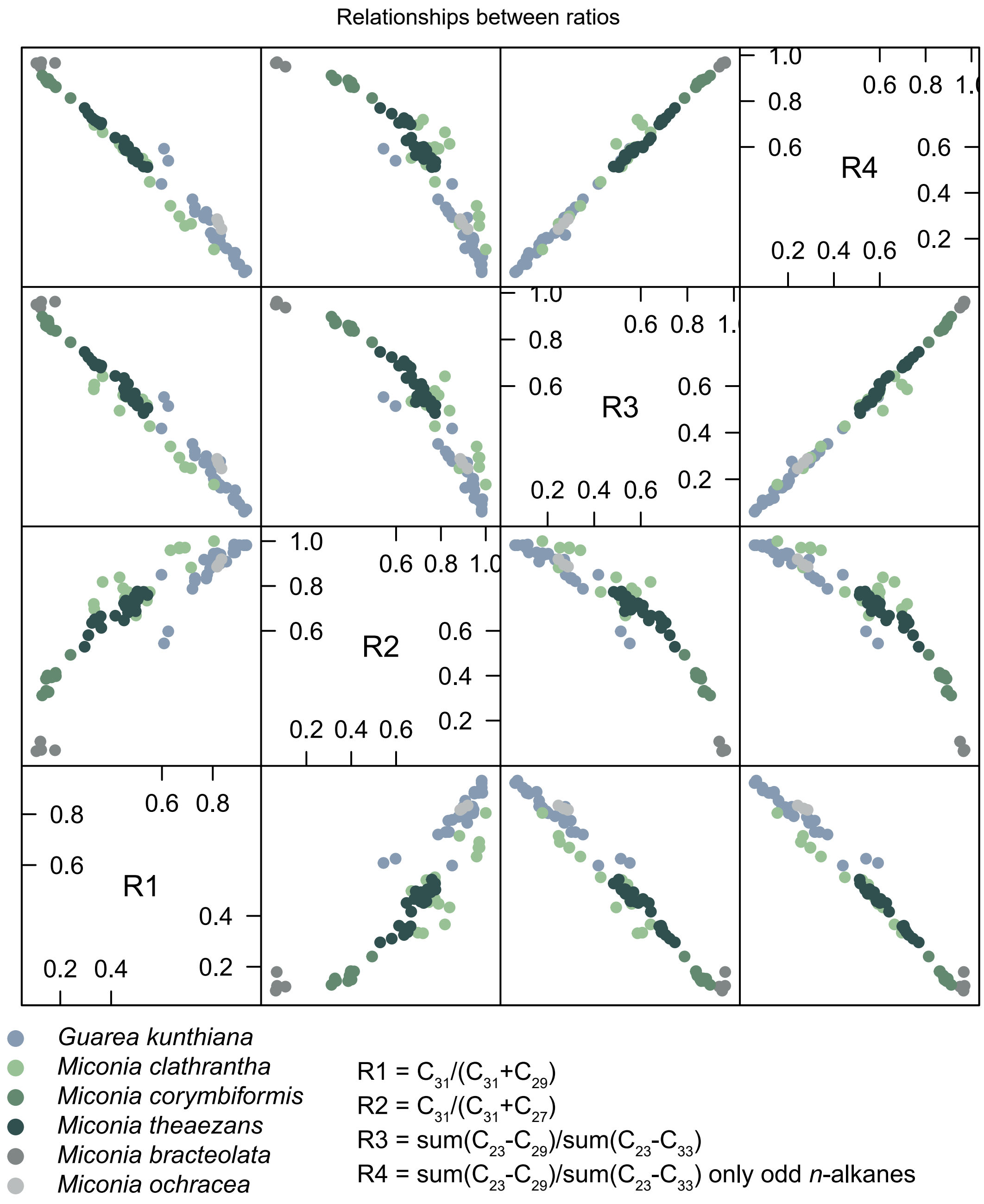
Relationships between ratios
R1
R2
R3
R4
0.2
0.4
0.6
0.8
1.0
Guarea kunthiana
Miconia clathrantha
Miconia corymbiformis
Miconia theaezans
Miconia bracteolata
Miconia ochracea
R1 = C31/(C31+C29)
R2 = C31/(C31+C27)
R3 = sum(C23-C29)/sum(C23-C33)
R4 = sum(C23-C29)/sum(C23-C33) only odd n-alkanes
