# Supplementary figures and images for: Leaf wax n‐alkane patterns of six tropical montane tree species show species‐specific environmental response
Source: Ecol Evol. 2019 Jul 21;9(16):9120–8. doi: 10.1002/ece3.5458 (PMC6706217; doi:10.1002/ece3.5458)

Pearson  
Correlation

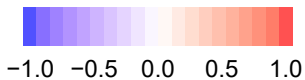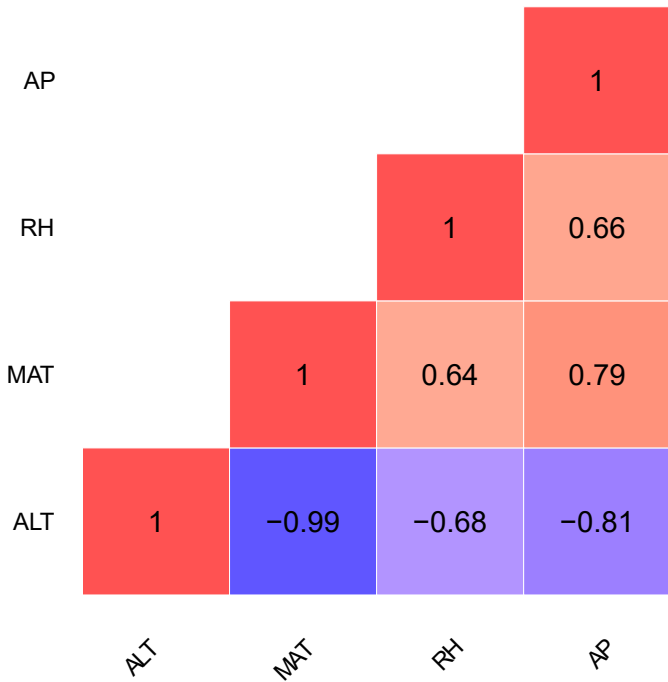

Supplement: Supplementary file 3 [file ECE3-9-9120-s003.pdf]
