# Supplementary material for: Leaf wax n‐alkane patterns of six tropical montane tree species show species‐specific environmental response
Source: Ecol Evol. 2019 Jul 21;9(16):9120–8. doi: 10.1002/ece3.5458 (PMC6706217; doi:10.1002/ece3.5458)
Supplement: Supplementary file 4 [file ECE3-9-9120-s004.docx]

| **Appendix 4** – Concentration and metrics variability of the replicate samples (Sample code). CONw = total n-alkane concentration (ng/g of dried sample); ACL = average chain length; RATIO = C31/(C31+C29); sd = standard deviation; CV(%) = coefficient of variance ; N(#) = number of replicates. | | | | | | | | | | |
| --- | --- | --- | --- | --- | --- | --- | --- | --- | --- | --- |
| **Sample code** | **CONw** | **±sd** | **CV (%)** | **ACL** | **±sd** | **CV (%)** | **RATIO** | **±sd** | **CV (%)** | **N(#)** |
| GK 03 | 141.2 | 72.9 | 51.6 | 30.9 | 0.1 | 0.4 | 0.9 | 0.0 | 2.8 | 2 |
| GK 12 | 124.8 | 61.3 | 49.1 | 30.9 | 0.1 | 0.3 | 0.9 | 0.0 | 4.4 | 2 |
| GK 13 | 78.1 | 6.10 | 7.90 | 29.3 | 0.1 | 0.3 | 0.6 | 0.0 | 1.9 | 2 |
| GK 15 | 147.8 | 26.0 | 17.6 | 30.7 | 0.0 | 0.2 | 0.8 | 0.0 | 0.7 | 3 |
| MCL 22 | 52.9 | 8.60 | 16.2 | 30.8 | 0.1 | 0.3 | 0.8 | 0.1 | 8.4 | 2 |
| MTH 17 | 290.6 | 28.6 | 9.80 | 29.1 | 0.0 | 0.1 | 0.4 | 0.0 | 2.3 | 3 |
