# Supplementary material for: Leaf wax n‐alkane patterns of six tropical montane tree species show species‐specific environmental response
Source: Ecol Evol. 2019 Jul 21;9(16):9120–8. doi: 10.1002/ece3.5458 (PMC6706217; doi:10.1002/ece3.5458)
Supplement: Supplementary file 5 [file ECE3-9-9120-s005.docx]

**Appendix 5** – Spearman’s rank correlation coefficients (r_s_) of the average chain length (ACL_23-33_) and the ratio (C_31_/(C_31_+C_29_)) against three environmental gradients: mean annual temperature (MAT), mean relative air humidity (RH) and mean annual precipitation (AP). Significance legend as follows: * p < 0.01, ** p <0.005, *** p < 0.001.

| **metric** | **variable** | **species** | **rs** | **p-value** |  |
| --- | --- | --- | --- | --- | --- |
|  |  | *Guarea kunthiana* | 0.44 | 0.021 |  |
|  |  | *Miconia clathrantha* | 0.50 | 0.020 |  |
|  | MAT | *Miconia corymbiformis* | 0.04 | 0.915 |  |
|  |  | *Miconia theaezans* | 0.37 | 0.068 |  |
|  |  | Total | 0.66 | 0.000 | *** |
|  |  | *Guarea kunthiana* | 0.50 | 0.008 | * |
|  |  | *Miconia clathrantha* | 0.06 | 0.805 |  |
| ACL | RH | *Miconia corymbiformis* | -0.04 | 0.915 |  |
| 23-33 |  | *Miconia theaezans* | 0.36 | 0.076 |  |
|  |  | Total | 0.44 | 0.000 | *** |
|  |  | *Guarea kunthiana* | 0.50 | 0.007 | * |
|  |  | *Miconia clathrantha* | -0.22 | 0.328 |  |
|  | AP | *Miconia corymbiformis* | 0.04 | 0.915 |  |
|  |  | *Miconia theaezans* | 0.39 | 0.056 |  |
|  |  | Total | 0.58 | 0.000 | *** |
|  |  |  |  |  |  |
|  |  | *Guarea kunthiana* | 0.44 | 0.021 |  |
|  |  | *Miconia clathrantha* | 0.56 | 0.009 | * |
|  | MAT | *Miconia corymbiformis* | 0.21 | 0.565 |  |
|  |  | *Miconia theaezans* | 0.58 | 0.003 | ** |
|  |  | Total | 0.61 | 0.000 | *** |
|  |  | *Guarea kunthiana* | 0.55 | 0.003 | ** |
|  |  | *Miconia clathrantha* | -0.02 | 0.930 |  |
| C_31_/ | RH | *Miconia corymbiformis* | -0.21 | 0.565 |  |
| (C_31_+C_29_) |  | *Miconia theaezans* | 0.28 | 0.180 |  |
|  |  | Total | 0.39 | 0.000 | *** |
|  |  | *Guarea kunthiana* | 0.57 | 0.002 | ** |
|  |  | *Miconia clathrantha* | -0.26 | 0.248 |  |
|  | AP | *Miconia corymbiformis* | 0.21 | 0.565 |  |
|  |  | *Miconia theaezans* | 0.23 | 0.274 |  |
|  |  | Total | 0.53 | 0.000 | *** |
