# Supplementary material for: Leaf wax n‐alkane patterns of six tropical montane tree species show species‐specific environmental response
Source: Ecol Evol. 2019 Jul 21;9(16):9120–8. doi: 10.1002/ece3.5458 (PMC6706217; doi:10.1002/ece3.5458)
Supplement: Supplementary file 6 [file ECE3-9-9120-s006.docx]

| **Appendix 6** (part I of II)– Spearman’s rank correlation coefficients (r_s_) of odd chain length *n*-alkanes from individual species and site total against three environmental gradients: mean annual temperature (MAT), mean relative air humidity (RH) and mean annual precipitation (AP). Significance legend as follows: * p < 0.01, ** p <0.005, *** p < 0.001 | | | | | |
| --- | --- | --- | --- | --- | --- |
| ***n* -alkane** | **variable** | **species** | **r_s_** | **p-value** |  |
|  |  | *Guarea kunthiana* | 0.55 | 0.003 | ** |
|  |  | *Miconia clathrantha* | 0.57 | 0.007 | * |
|  | MAT | *Miconia corymbiformis* | 0.10 | 0.775 |  |
|  |  | *Miconia theaezans* | 0.53 | 0.007 | * |
|  |  | Total | 0.61 | 0.000 | *** |
|  |  | *Guarea kunthiana* | 0.58 | 0.002 | ** |
|  |  | *Miconia clathrantha* | 0.05 | 0.841 |  |
| C33 | RH | *Miconia corymbiformis* | -0.10 | 0.775 |  |
|  |  | *Miconia theaezans* | 0.31 | 0.129 |  |
|  |  | Total | 0.38 | 0.000 | *** |
|  |  | *Guarea kunthiana* | 0.58 | 0.002 | ** |
|  |  | *Miconia clathrantha* | -0.18 | 0.448 |  |
|  | AP | *Miconia corymbiformis* | 0.10 | 0.775 |  |
|  |  | *Miconia theaezans* | 0.23 | 0.264 |  |
|  |  | Total | 0.47 | 0.000 | *** |
|  |  |  |  |  |  |
|  |  | *Guarea kunthiana* | 0.05 | 0.798 |  |
|  |  | *Miconia clathrantha* | 0.63 | 0.002 | ** |
|  | MAT | *Miconia corymbiformis* | 0.18 | 0.616 |  |
|  |  | *Miconia theaezans* | 0.32 | 0.118 |  |
|  |  | Total | 0.56 | 0.000 | *** |
|  |  | *Guarea kunthiana* | 0.22 | 0.271 |  |
|  |  | *Miconia clathrantha* | -0.03 | 0.883 |  |
| C31 | RH | *Miconia corymbiformis* | -0.18 | 0.616 |  |
|  |  | *Miconia theaezans* | 0.39 | 0.051 |  |
|  |  | Total | 0.35 | 0.000 | *** |
|  |  | *Guarea kunthiana* | 0.31 | 0.114 |  |
|  |  | *Miconia clathrantha* | -0.31 | 0.169 |  |
|  | AP | *Miconia corymbiformis* | 0.18 | 0.616 |  |
|  |  | *Miconia theaezans* | 0.36 | 0.075 |  |
|  |  | Total | 0.49 | 0.000 | *** |
|  |  |  |  |  |  |
|  |  | *Guarea kunthiana* | -0.44 | 0.022 |  |
|  |  | *Miconia clathrantha* | -0.45 | 0.042 |  |
|  | MAT | *Miconia corymbiformis* | -0.42 | 0.233 |  |
|  |  | *Miconia theaezans* | -0.82 | 0.000 | *** |
|  |  | Total | -0.47 | 0.000 | *** |
|  |  | *Guarea kunthiana* | -0.54 | 0.004 | ** |
|  |  | *Miconia clathrantha* | 0.05 | 0.839 |  |
| C29 | RH | *Miconia corymbiformis* | 0.42 | 0.233 |  |
|  |  | *Miconia theaezans* | -0.17 | 0.419 |  |
|  |  | Total | -0.38 | 0.000 | *** |
|  |  | *Guarea kunthiana* | -0.55 | 0.003 | ** |
|  |  | *Miconia clathrantha* | 0.28 | 0.212 |  |
|  | AP | *Miconia corymbiformis* | -0.42 | 0.233 |  |
|  |  | *Miconia theaezans* | -0.15 | 0.480 |  |
|  |  | Total | -0.39 | 0.000 | *** |
|  | <0.01 * ; <0.005**; <0.001*** | |  |  |  |

| **Appendix 6** (part II of II)– Spearman’s rank correlation coefficients (r_s_) of odd chain length *n*-alkanes from individual species and site total against three environmental gradients: mean annual temperature (MAT), mean relative air humidity (RH) and mean annual precipitation (AP). Significance legend as follows: * p < 0.01, ** p <0.005, *** p < 0.001 | | | | | |
| --- | --- | --- | --- | --- | --- |
| ***n* -alkane** | **variable** | **species** | **r_s_** | **p-value** |  |
|  |  | *Guarea kunthiana* | -0.40 | 0.041 |  |
|  |  | *Miconia clathrantha* | -0.61 | 0.003 | ** |
|  | MAT | *Miconia corymbiformis* | 0.14 | 0.694 |  |
|  |  | *Miconia theaezans* | -0.51 | 0.010 | * |
|  |  | Total | -0.67 | 0.000 | *** |
|  |  | *Guarea kunthiana* | -0.45 | 0.018 |  |
|  |  | *Miconia clathrantha* | -0.77 | 0.000 | *** |
| C27 | RH | *Miconia corymbiformis* | -0.14 | 0.694 |  |
|  |  | *Miconia theaezans* | -0.53 | 0.006 | * |
|  |  | Total | -0.47 | 0.000 | *** |
|  |  | *Guarea kunthiana* | -0.45 | 0.018 |  |
|  |  | *Miconia clathrantha* | -0.45 | 0.041 |  |
|  | AP | *Miconia corymbiformis* | 0.14 | 0.694 |  |
|  |  | *Miconia theaezans* | -0.51 | 0.010 | * |
|  |  | Total | -0.58 | 0.000 | *** |
|  |  |  |  |  |  |
|  |  | *Guarea kunthiana* | -0.40 | 0.037 |  |
|  |  | *Miconia clathrantha* | -0.40 | 0.070 |  |
|  | MAT | *Miconia corymbiformis* | 0.47 | 0.174 |  |
|  |  | *Miconia theaezans* | 0.76 | 0.000 | *** |
|  |  | Total | -0.56 | 0.000 | *** |
|  |  | *Guarea kunthiana* | -0.27 | 0.179 |  |
|  |  | *Miconia clathrantha* | -0.45 | 0.041 |  |
| C25 | RH | *Miconia corymbiformis* | -0.47 | 0.174 |  |
|  |  | *Miconia theaezans* | 0.31 | 0.138 |  |
|  |  | Total | -0.44 | 0.000 | *** |
|  |  | *Guarea kunthiana* | -0.27 | 0.172 |  |
|  |  | *Miconia clathrantha* | -0.18 | 0.424 |  |
|  | AP | *Miconia corymbiformis* | 0.47 | 0.174 |  |
|  |  | *Miconia theaezans* | 0.21 | 0.314 |  |
|  |  | Total | -0.58 | 0.000 | *** |
|  |  |  |  |  |  |
|  |  | *Guarea kunthiana* | -0.24 | 0.231 |  |
|  |  | *Miconia clathrantha* | 0.10 | 0.667 |  |
|  | MAT | *Miconia corymbiformis* | -0.32 | 0.361 |  |
|  |  | *Miconia theaezans* | 0.14 | 0.516 |  |
|  |  | Total | -0.53 | 0.000 | *** |
|  |  | *Guarea kunthiana* | -0.20 | 0.319 |  |
|  |  | *Miconia clathrantha* | 0.09 | 0.692 |  |
| C23 | RH | *Miconia corymbiformis* | 0.32 | 0.361 |  |
|  |  | *Miconia theaezans* | 0.55 | 0.004 | ** |
|  |  | Total | -0.31 | 0.000 | *** |
|  |  | *Guarea kunthiana* | -0.29 | 0.145 |  |
|  |  | *Miconia clathrantha* | 0.27 | 0.234 |  |
|  | AP | *Miconia corymbiformis* | -0.32 | 0.361 |  |
|  |  | *Miconia theaezans* | 0.17 | 0.423 |  |
|  |  | Total | -0.52 | 0.000 | *** |
|  | <0.01 * ; <0.005**; <0.001*** | |  |  |  |
